# Aerobic Training for Obesity Management in Individuals with Down Syndrome: A Bibliometric and Meta-Analyses

**DOI:** 10.3390/healthcare14081052

**Published:** 2026-04-15

**Authors:** Sieun Park, Seung Kyum Kim

**Affiliations:** 1 Department of Sports Science, Seoul National University of Science and Technology, Seoul 01811, Republic of Korea; parksy24510294@seoultech.ac.kr; 2 Institute of Sports Science, Seoul National University of Science and Technology, Seoul 01811, Republic of Korea

**Keywords:** down syndrome, physical activity, obesity, aerobic training

## Abstract

**Highlights:**

**What are the main findings?**

- Research on Down syndrome and physical activity has increased significantly, with “obesity” emerging as the most rapidly growing and central topic.
- Aerobic training significantly improves body composition—specifically fat mass and waist circumference—in individuals with Down syndrome and obesity.

**What are the implications of the main findings?**

- Weight-based metrics (e.g., body weight) may not adequately capture the benefits of exercise in individuals with Down syndrome.
- Aerobic training can be considered a primary non-pharmacological strategy for obesity management in Down syndrome.

**Abstract:**

**Background/Objectives**: Down syndrome (DS), the most common chromosomal disorder, is associated with obesity and related metabolic complications. Although physical activity (PA) improves health outcomes in individuals with DS, global research trends in this field have not been systematically synthesized, and evidence regarding the effects of aerobic training (AT) on obesity-related parameters in individuals with DS remains inconsistent. This study incorporated a dual bibliometric and meta-analytical approach. **Methods**: First, the bibliometric analysis included 321 original research articles published between 2001 and 2024, retrieved from Scopus, Web of Science, and PubMed. Second, a meta-analysis of 15 randomized controlled trials (n = 477) was conducted to examine the effects of AT on obesity-related parameters, including body weight (BW), body mass index (BMI), fat mass (FM), waist circumference (WC), and waist-to-hip ratio (WHR) in individuals with DS. **Results**: Keyword co-occurrence and collaboration network analyses revealed a notable increase in research output since 2018, with “adolescent,” “obesity,” and “intellectual disability” the most co-occurring keywords associated with DS and PA. “Obesity” emerged as the most prominently growing keyword associated with DS and PA. A meta-analysis concluded that AT reduced FM (standardized mean differences [SMD] = −0.44; *p* < 0.001) and WC (SMD = −0.39; *p* < 0.01), while subtle changes in BW, BMI, and WHR were found. These findings suggest that AT improves body composition, particularly reducing central adiposity, even without changes in traditional weight-based metrics. **Conclusions**: Our findings demonstrate that AT can be an effective non-pharmacological strategy for improving body composition in individuals with DS and obesity and highlight the urgent need to shift clinical and research paradigms toward multidimensional, individualized health strategies that support PA and healthy body composition throughout the lifespan.

## 1. Introduction

Down syndrome (DS), caused by trisomy of chromosome 21, is the most common chromosomal abnormality, affecting approximately 1 in 400–1500 live births worldwide [1,2]. Individuals with DS exhibit a broad spectrum of phenotypic characteristics, including intellectual disabilities, motor impairments, and various comorbidities, such as congenital heart defects and endocrine disorders [3,4]. Although advances in medical care have extended the life expectancy of individuals with DS from approximately 9–12 years in the early to mid-20th century to over 60 years today, this has not necessarily translated into an improved quality of life [4].

Obesity is one of the most prevalent health issues among individuals with DS. The prevalence of overweight and obesity in this population is much higher than that in individuals without DS, with an estimated prevalence of >45% and pediatric rates as high as 47.8% [5,6]. This can be attributed to a combination of various factors, such as low basal metabolic rate, hypotonia, short stature, cognitive limitations, poor dietary habits, hormonal imbalances, and reduced physical activity [7,8]. Obesity can exacerbate metabolic complications, including insulin resistance, dyslipidemia, and cardiovascular diseases in individuals with DS, and has been linked to the early onset of neurodegenerative conditions, such as Alzheimer’s disease [3,9].

Additionally, sarcopenic obesity, a condition characterized by the simultaneous presence of low muscle mass and excess fat mass, occurs more frequently and severely in individuals with DS [10,11]. Studies indicate that adults with DS have a significantly higher prevalence of sarcopenia (35.6% vs. 19.9% without DS) and that approximately 36% of young adults meet the sarcopenia criteria [11], exacerbating metabolic efficiency and complicating traditional weight management strategies for individuals with DS [7,12].

Accumulating data indicate that physical activity (PA) can be used as a non-pharmacological intervention for managing obesity and improving health outcomes, even in individuals with DS [13,14]. Aerobic training (AT) has been shown to have the most beneficial effects on cardiovascular function, fat oxidation, and cognitive performance [12,15]. Nevertheless, individuals with DS often face barriers to engaging in PA, possibly because of hypotonia, low motivation, motor planning deficits, joint laxity, balance and coordination challenges, and limited access to inclusive exercise programs [7,16]. These barriers can lead to a persistent pattern of physical inactivity, beginning in childhood and extending into adulthood [17,18]. In addition, the results remain limited and inconsistent owing to variations in study design, small sample sizes, short intervention durations, and diverse outcome measures [13,19]. Although some studies have reported potential benefits across various physical and psychosocial domains, consistent improvements have been observed, primarily in work-related performance measures [20,21]. In the broader literature on individuals with intellectual disabilities, a relatively greater number of reviews have demonstrated the positive effects of PA for them; however, only a small proportion have examined DS as a distinct subgroup, and even fewer have specifically investigated the impact of AT on obesity-related outcomes in this population [13,22].

To address these gaps, this study used a two-pronged approach. First, a quantitative bibliometric analysis was performed to identify research trends, emerging themes, and research networks in the field of DS and PA research. From this analysis, “obesity” emerged as the most prominently growing keyword associated with DS and PA. Furthermore, we did not find a previous study which systemically and statistically investigated the effect of AT in individuals with both DS and obesity. Therefore, a meta-analysis was conducted to assess the effects of AT, known to be most effective for weight management, on obesity-related factors, including body mass index (BMI), fat mass (FM), waist circumference (WC), and waist-to-hip ratio (WHR), in individuals with DS classified as obese. Through these complementary approaches, this study provides an updated synthesis of the current evidence and aims to inform the development of targeted and effective exercise interventions to prevent and manage obesity in individuals with DS.

## 2. Methods

### 2.1. Bibliometric Analysis Method

**Figure 1:**
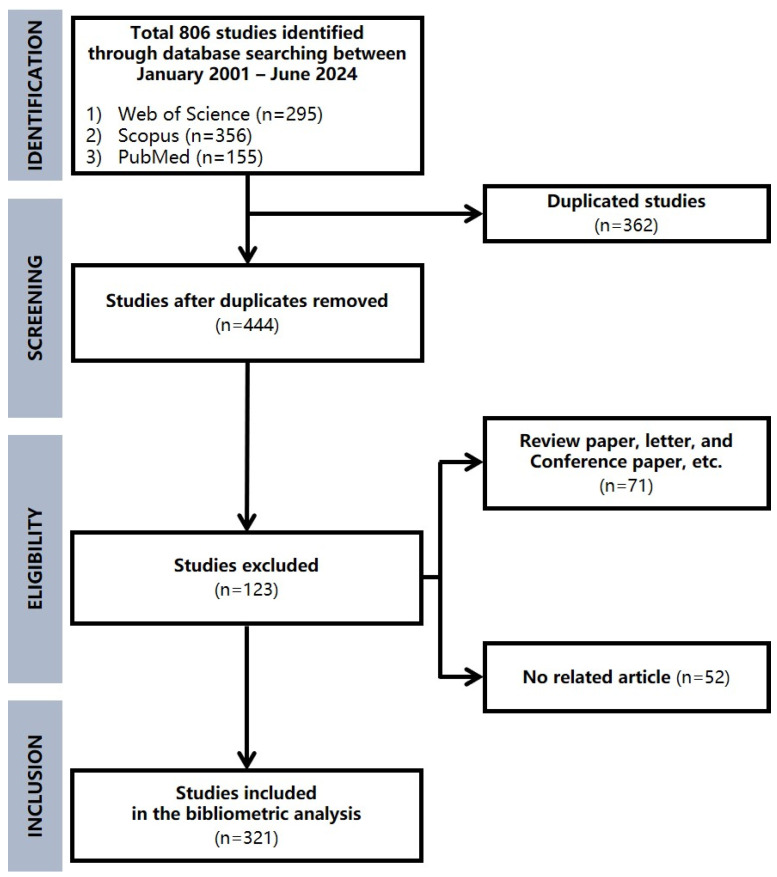
Flowchart of bibliometric analysis selection process.

Articles containing the two keywords, “Down syndrome” (including “trisomy 21” and “Down’s syndrome”) and “physical activity” (including “exercise,” “exercise training,” “physical training,” “walking,” “running,” “physical performance,” and “physical fitness”) were searched for in Web of Science, Scopus, and PubMed on 1 August 2025. As there were fewer than five publications in 2000, we only included articles published between January 2001 and December 2024 in the analysis. The initial searches identified 806 articles (295 from Web of Science, 356 from Scopus, and 155 from PubMed). After removing duplicates, 444 articles remained (Table 1). Non-original articles, such as reviews and conference proceedings, and non-human subject studies (n = 52) were excluded, and a final set of 321 original research articles met the inclusion criteria for bibliometric analysis (Figure 1). The keywords extracted from the final publications were reviewed by two researchers (S.P. and S.K.K.) and standardized, and similar words were examined to unify the terms between the evaluators. Keyword co-occurrence, co-authorship, and collaboration networks were analyzed using VOSviewer (v1.6.19) [23].

### 2.2. Meta-Analysis Method

Because the bibliometric results revealed that “obesity” was the most co-occurring as well as the prominently growing co-occurring physiological keyword with DS and PA, we conducted a meta-analysis to study the effect of PA on obesity-related outcomes in individuals with DS from January 2001 to December 2024 (the same period as the bibliometric analysis). To the best of our knowledge, no previous meta-analysis has specifically examined PA effects on individuals with DS and obesity. In particular, we chose AT, as it has been widely recommended for obesity management and applied to individuals with DS and obesity [12,13]. Furthermore, no intervention studies were found for the effect of resistance training or flexibility exercises on this population. The review protocol was registered at PROSPERO (CRD420261304004). Our meta-analysis adhered to the Preferred Reporting Items for Systematic Reviews and Meta-Analyses (PRISMA) guidelines and applied the PICO framework [24].

(1) Population: Clinically diagnosed individuals with Down syndrome classified as obese (BMI ≥ 30 for adults; ≥85th percentile for children/adolescents) [25].
(2) Intervention: AT (e.g., walking, swimming, cycling, running) is either a standalone program or a major component of a multifaceted intervention. Interventions lasted for at least eight weeks, as this duration is the minimally recommended period to observe meaningful physiological adaptations and health benefits [26].
(3) Comparison: Control groups without exposure to AT included passive (no intervention), active (alternative exercise), and non-exercise-education-focused controls.
(4) Outcomes: Changes in BW, BMI, FM, WC, and WHR.
(5) Study Design: Only randomized controlled trials (RCTs) written in English were included.

**Figure 2:**
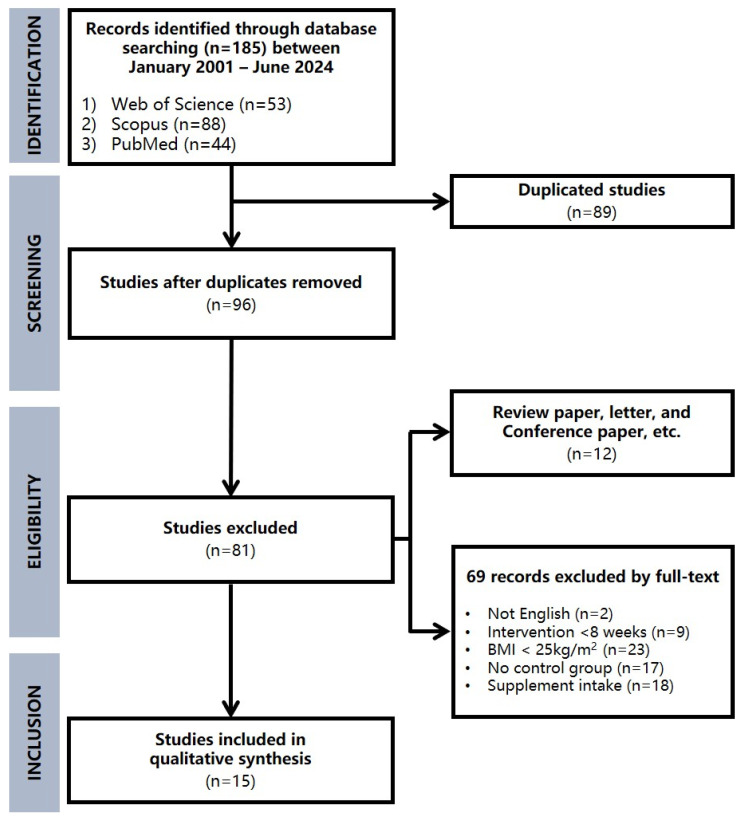
PRISMA flowchart for the study identification procedure of the meta-analysis.

The search strategy combined terms: (“Down syndrome” OR “trisomy 21” OR “Down’s syndrome”) AND (“obesity” OR “obese” OR “adiposity”) AND (“aerobic training” OR “endurance training” OR “aerobic exercise” OR “endurance exercise” OR “walking” OR “running” OR “cardio training” OR “cardio exercise”). The search included terms in the title, abstract and author keywords. The initial searches were conducted on 1 September 2025, and identified 185 articles (53 from Web of Science, 88 from Scopus, and 44 from PubMed). After removing duplicates (n = 89) and applying the eligibility criteria, 15 RCTs remained for further quantitative analysis (Figure 2).

### 2.3. Data Extraction and Quality Assessment

Two independent investigators (S.P. and S.K.K.) extracted data on the following characteristics: publication year, country, participant characteristics (age, sex, and sample size), intervention details, control conditions, outcome measures, pre- and post-means, and standard deviations. Disagreements were resolved by consensus. The methodological quality of the included studies in the meta-analysis was evaluated using the Physiotherapy Evidence Database (PEDro) scale, which has been validated to assess RCTs in rehabilitation research, evaluating internal validity and statistical interpretability [27]. The PEDro score ranges from 0 to 10, with a score < 4 indicating poor quality. Publication bias was checked using funnel plot asymmetry and Egger’s test, although its interpretation is limited due to the small number of included studies (n < 10) (Supplementary Figure S1).

### 2.4. Statistical Analysis

For the meta-analysis, standardized mean differences (SMDs) were calculated for all outcomes using Hedges’ g test with 95% confidence intervals, which are recommended for small sample sizes [28]. Effect sizes were interpreted according to Cohen’s guidelines: small (0.20–0.49), moderate (0.50–0.79), or large (≥0.80) [29]. Heterogeneity was assessed using the I^2^ statistic and categorized as low (~25%), moderate (~50%), or high (~75%) [30]. Random-effects models were used to account for clinical and methodological heterogeneity among the studies [31]. All analyses were executed with R software (version 3.4.4 for Windows) using the meta and metafor packages [32,33].

## 3. Results

### 3.1. Bibliometric Analysis Results

**Figure 3:**
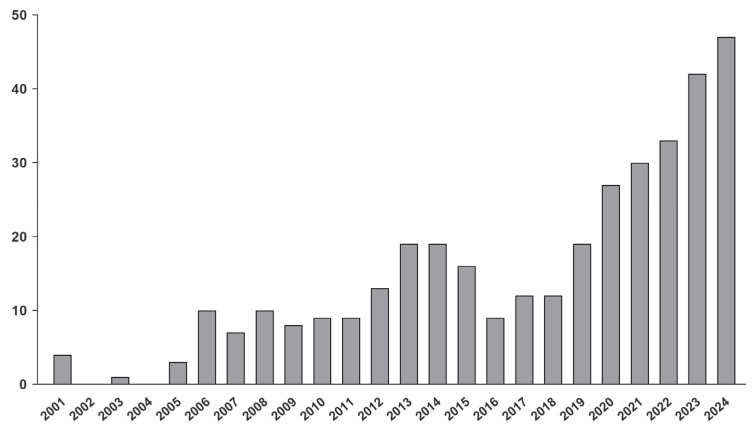
The annual number of publications having the two keywords “down syndrome” and “physical activity”.

A total of 321 original articles containing the keywords “DS” and “PA” were identified between January 2001 and December 2024. Figure 3 shows the annual number of publications, with a gradual increase in the number of studies. Before 2005, fewer than five papers were published each year; however, a sharp rise was observed starting in 2018, indicating increased academic interest and research activity on this topic.

A total of 1009 unique authors contributed to these publications. Most papers (94.4%) involved collaboration between three or more authors, reflecting a high degree of research collaboration. Ordonez F.J. and Rosety R.M. from Spain were the most productive authors (n = 20), displaying the strongest research network (link strength = 62), followed by Fernhall B. from the USA (Table 2).

**Figure 4:**
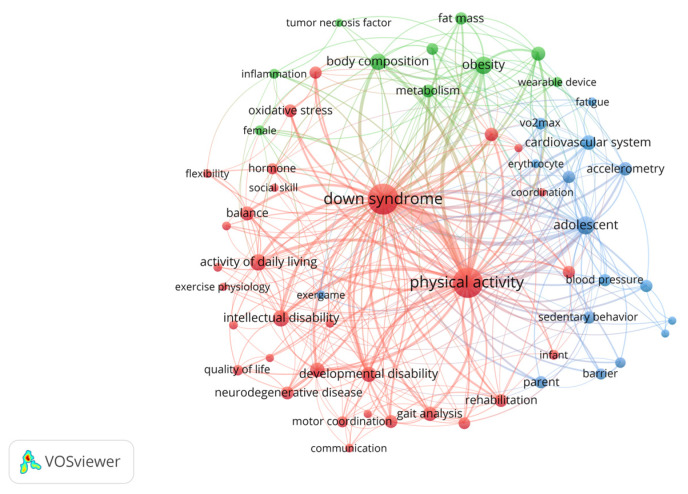
Network visualization map for the co-occurrence of keywords from original articles with the keywords “down syndrome” and “physical activity” published between January 2000 and December 2024. The similarity between two keywords is proportional to the number of simultaneous occurrences, placing words with high similarity close together. The size of the circles representing keywords increases with the relevance of the keywords (total link strength), and the distinctive colors of the circles and lines mean the difference in modularity.

Keyword analysis identified 20 frequently co-occurring terms with DS and PA (Table 3). The most prevalent keywords were “adolescent,” “obesity,” “intellectual disability,” “body composition,” and “activities of daily living.” Figure 4 illustrates a network visualization of these keywords, displaying three primary thematic clusters: a green cluster encompassing obesity, body composition, and fat mass, highlighting the focus on metabolic health and nutritional factors; a red cluster centered on intellectual and developmental disability, reflecting functional limitations and neural defects; and a blue cluster covering the cardiovascular system and blood pressure, indicating research interest in cardiorespiratory fitness, VO_2_max, and overall cardiovascular health.

To classify changes in research trends by era, keywords were grouped by decade (2000–2009, 2010–2019, and 2020–2024), as shown in Table 4. From 2000 to 2009, studies predominantly focused on physiological markers, including oxidative stress, hormone levels, and basic metabolic characteristics, reflecting an emphasis on understanding biological mechanisms. From 2010 to 2019, a clear shift was observed due to the investigation of cognitive function, behavioral adaptation, and daily living skills, mirroring the growing recognition of neuropsychological development and quality of life as central research topics. Since 2020, research interest has been focused on lifestyle factors, such as dietary patterns, sedentary behavior, and daily physical activity. This recent trend highlights the expanded focus on real-world health promotion, prevention strategies, and holistic well-being. Across eras, these evolving themes indicate an ongoing transition from reductionist, biologically driven research to more comprehensive, multidimensional approaches that consider physical, cognitive, and behavioral domains in the study of DS and PA. Based on these results, we selected “obesity” for the meta-analysis to comprehensively evaluate the efficacy of AT on obese DS patients, which was revealed by bibliometric analysis to be a significantly increasing topic in recent years.

### 3.2. Meta-Analysis Results

#### 3.2.1. Quality Check

PEDro scores ranged from 4 to 8 points, with a mean score of 5.71, suggesting moderate methodological quality (Table 5). All studies fulfilled the following four criteria: random allocation, groups similar at baseline, adequate follow-up, and between-group differences reported. None of the studies met the criteria for participant or therapist blinding.

#### 3.2.2. Study Characteristics

The characteristics of the 15 RCTs are summarized in Table 6. The total sample size was 477 participants (intervention group, n = 197; control group, n = 280), with ages ranging from 14 to 39 years. Most AT interventions lasted 8–12 weeks, with a typical training frequency of 2–3 sessions per week. Common aerobic modalities included treadmill walking, swimming, cycling, and playing active video games. The obesity-associated variables in these studies included one or more of the following parameters: BW, BMI, FM, WC, and WHR (Table 6).

#### 3.2.3. Body Weight and BMI

**Figure 5:**
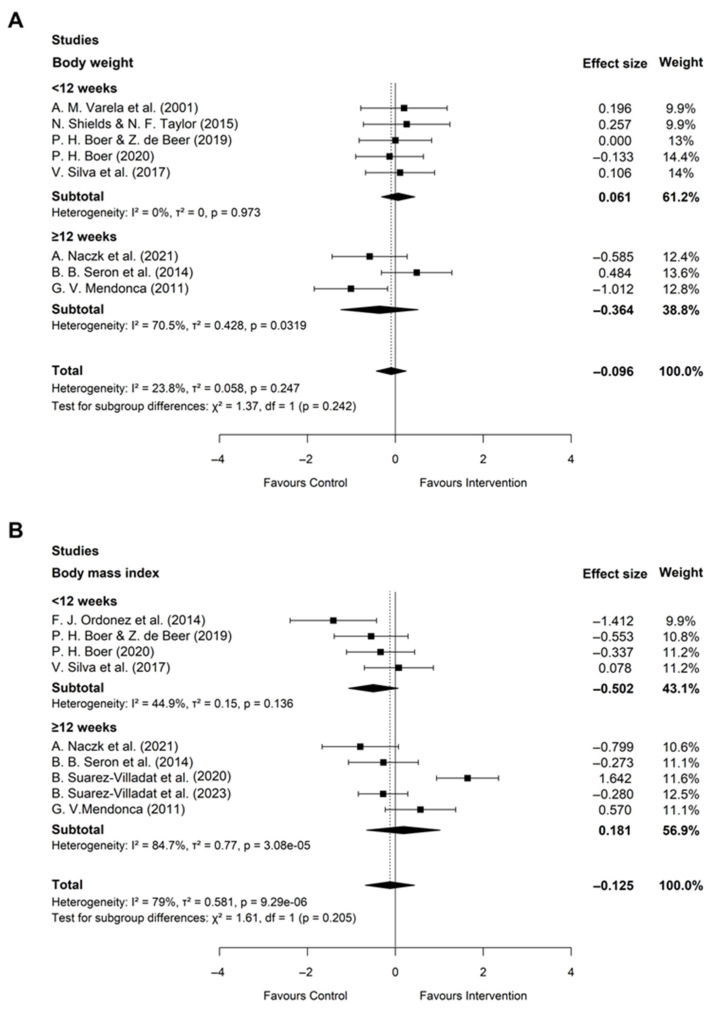
Forest plot of the effect of aerobic training versus control on body weight (**A**) and body mass index (**B**) in obese individuals with Down syndrome. Diamonds indicate pooled effect sizes [34,39,40,41,42,44,45,46,47,48].

Eight studies (n = 179) assessed changes in BW (Figure 5A). AT did not result in a significant effect on BW (standardized mean difference [SMD] = −0.10; 95% confidence interval [CI]: −0.44 to 0.25; *p* = 0.58) and was characterized by low heterogeneity (I^2^ = 23.8%) (Figure 5A). A subgroup analysis by training duration (less than 12 weeks vs. more than 12 weeks) was further performed, and no significant difference was found (*p* = 0.97).

Nine studies (n = 261) included BMI measurements, showing no significant effect of AT (SMD = −0.13; 95% CI: −0.69 to 0.44; *p* = 0.66) and substantial heterogeneity among the studies (I^2^ = 79.0%) (Figure 5B). A subgroup difference was also not found (*p* = 0.21). This finding suggests that AT may not substantially influence the BW of individuals with DS and obesity, potentially because of concurrent increases in lean mass or limitations in weight as a measure of body composition. Furthermore, a relatively small sample size could also be a possible reason for there being no benefit to these weight-variables.

#### 3.2.4. Fat Mass

**Figure 6:**
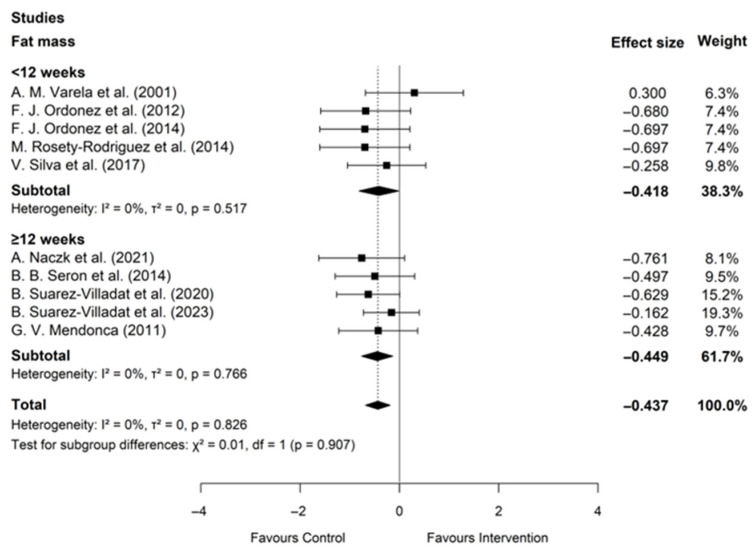
Forest plot of the effect of aerobic training (AT) versus control on fat mass (FM) in obese individuals with Down syndrome. Diamonds indicate pooled effect sizes. An effect size smaller than zero favors AT for FM [34,36,37,38,39,40,42,45,47,48].

Ten studies (n = 248) reported post-intervention FM. AT resulted in a statistically significant reduction in FM (SMD = −0.44; 95% CI: −0.68 to −0.19; *p* < 0.001), with no substantial heterogeneity (I^2^ = 0%) (Figure 6). Additionally, a subgroup difference by duration was not found (*p* = 0.91). This finding highlights the consistent efficacy of AT in reducing fat mass in individuals with DS, supporting its role as an effective intervention for improving body composition in this population. Also, considering adherence concerns in this population, AT for less than 12 weeks might be enough to induce FM reduction.

#### 3.2.5. Waist Circumference and Waist-to-Hip Ratio

**Figure 7:**
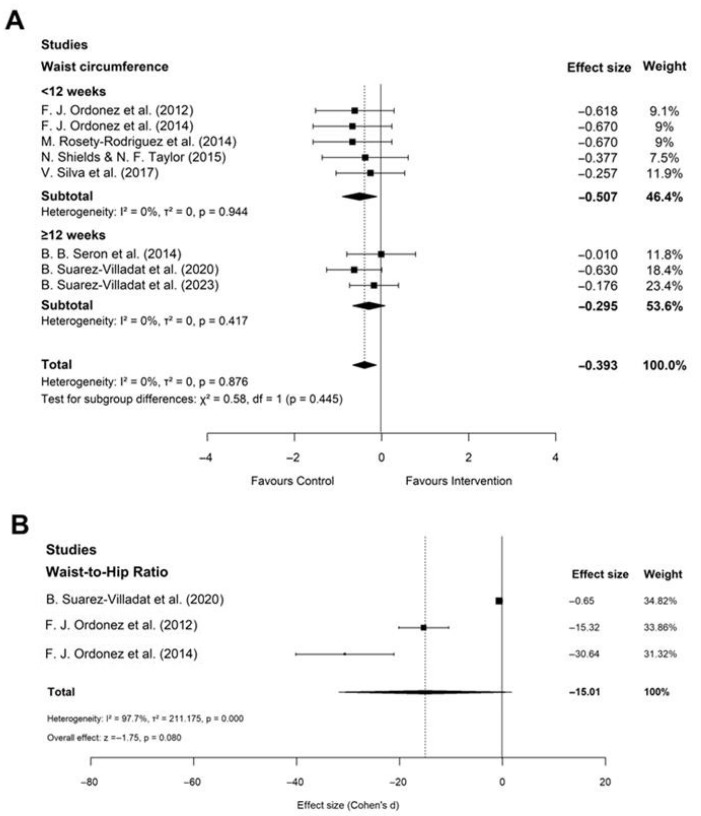
Forest plot of the effect of aerobic training (AT) versus control on waist circumference (WC) (**A**) and waist-to-hip ratio (WHR) (**B**) in obese individuals with Down syndrome. Diamonds indicate pooled effect sizes. An effect size smaller than zero favors AT for WC [34,38,39,40,41,42,45,48].

The pooled analysis of eight studies (n = 221) showed a significant reduction in WC following AT (SMD = −0.39; 95% CI: −0.66 to −0.12; *p* < 0.01), with minimal heterogeneity (I^2^ = 0%) (Figure 7A). This consistent decrease suggests that AT is particularly effective in reducing central adiposity, a key risk factor for metabolic and cardiovascular complications in individuals with DS. A subgroup analysis also indicates no difference by training duration (*p* = 0.45), suggesting that AT for less than 12 weeks can be effective in reducing central adiposity for this population. Analysis of data from three studies (n = 85) yielded a negative, though non-significant, trend for WHR (SMD = −0.39; 95% CI: −0.80 to 0.03; *p* = 0.08) with very high heterogeneity (I^2^ = 97.7%) (Figure 7B). Publication bias was also detected for WHR (Supplementary Figure S1), although this result should be interpreted with caution given the low statistical power and small number of studies. This suggests that the potential effect of AT on WHR in individuals with DS remains inconclusive.

## 4. Discussion

This study provides a comprehensive synthesis of the evolving research landscape in DS and PA research and the quantitative impact of AT on obesity-related measurements in individuals with DS. Through a two-pronged approach involving bibliometric and meta-analytical techniques, our findings offer novel insights into research trends on this topic and the efficacy of AT on the body composition of individuals with DS and obesity. The bibliometric analysis demonstrated a notable expansion of research on DS and PA over the past two decades, identifying the keyword “obesity” as a rapidly growing focus in recent years. The meta-analysis, based on 15 RCTs, revealed that AT significantly reduced FM and WC, but showed subtle effects on BW, BMI, or WHR.

Consistent with previous literature, our bibliometric analysis revealed a growing scientific interest in DS and PA, particularly in recent years. The emergence of obesity as the most widely studied physiological keyword reflects concerns regarding the high prevalence of metabolic complications associated with obesity in individuals with DS. In recent years, the emphasis on physiological markers of metabolic and cardiovascular health has broadened to include cognitive function, nutritional habits, and lifestyle factors, reflecting a growing appreciation for the complex, multifactorial nature of health in DS [17,22].

The meta-analysis in this study demonstrated that AT can exert benefits on specific obesity-related parameters in individuals with DS, despite some limitations in traditional metrics such as BW and BMI. The AT-induced reduction in FM is consistent with previous studies showing that exercise can mitigate central adiposity, even in the absence of weight loss [15,49]. These effects are clinically meaningful because excess visceral fat is implicated in insulin resistance, cardiovascular risk, and systemic inflammation in individuals with DS [5,8]. In contrast, the absence of reductions in BW and BMI may reflect the limited sensitivity of these anthropometric measures to subtle but meaningful changes in body composition, particularly in clinical populations prone to low muscle mass and high FM such as those with DS [7,11]. Additionally, previous studies have shown that prevalence of metabolic syndrome and dyslipidemia in individuals with DS is comparable to BMI- matched non-DS populations; however, when central obesity is pronounced, such metabolic disease rates rise sharply [50,51]. Therefore, our results are still meaningful in that AT-induced reductions in FM and WC without changes in BW and BMI can contribute to preventing or alleviating metabolic diseases.

There was a trend toward a reduction in WHR following AT (SMD = −0.39, *p* = 0.08). However, it was noticed that these results are from the small number of studies (n = 3) and have significant between-study heterogeneity (I^2^ = 97.72%). Although WHR is a marker of fat distribution, its variability may reflect differences in participant characteristics, measurement protocols, or intervention duration [44,52,53] and, thus, support the need for future studies exploring the effect of AT on this crucial factor with a standardized approach.

It is noteworthy that the methodological quality of the included RCTs was moderate and that most interventions lasted 8–12 weeks, with 2–3 sessions per week, reflecting real-world feasibility and adherence concerns in this population. However, variability in intervention protocols, such as training modality and intensity, cannot be generalized. Additionally, the limited number of studies (n = 3–10) and the absence of lean body mass or sarcopenia-specific outcomes constitute important gaps in the existing literature. Although the focus of this meta-analysis was on AT, the limited available evidence suggests that such interventions could contribute to a modest improvement or the preservation of lean body mass, potentially mitigating sarcopenic risk. However, the lack of consistent reporting on muscle-specific outcomes precludes conclusions for the efficacy of AT on muscle mass and function. In addition, the lack of blinding and random allocation in some trials raises the possibility of bias [54]. Finally, the absence of long-term follow-up data limits inferences regarding the durability of the observed benefits.

Therefore, future research should prioritize the investigation of multimodal interventions that integrate aerobic and resistance training, thereby targeting both fat reduction and muscle preservation as well as sarcopenic risk prevention. Outcome assessments should routinely include lean mass, inflammatory markers, and cognitive function to better capture the multifaceted benefits of PA in patients with DS [11,55]. Standardization of intervention protocols and reporting is essential to facilitate cross-study comparisons. There is also a need for research to be conducted in resource-limited settings and among understudied age groups, including older adults with DS.

## 5. Conclusions

This study is the first to comprehensively synthesize bibliometric and meta-analytical methods to evaluate the effectiveness of PA, particularly AT, in individuals with DS, emphasizing obesity-related outcomes. Bibliometric analysis has revealed a marked increase in PA and obesity research activity in DS over the past decade. A meta-analysis demonstrated that AT results in reductions in FM and WC, which are key predictors of metabolic risk, even though there are subtle changes in overall BW or BMI. Given the unique metabolic and physiological profiles of individuals with DS, such as sarcopenic obesity and impaired fat oxidation, efforts to reduce central adiposity and improve body composition should be prioritized in health management plans for this population. In addition, considering the methodological limitations and absence of long-term follow-up data, future studies are warranted to assess the combined effects of aerobic and resistance training and expand the outcome evaluation to include cognitive, psychosocial, and functional endpoints. This study provides evidence that AT can effectively reduce obesity-related metabolic risk in individuals with DS. These findings highlight the urgent need to shift clinical and research paradigms toward multidimensional, individualized health strategies that support physical activity and healthy body composition throughout the lifespan of individuals with DS.

## Figures and Tables

**Table 1:** Type of publications retrieved from January 2001 to December 2024.

Type of Paper	Frequency	%
Article	373	84
Review	40	9
Proceedings paper	9	2
Conference paper	9	2
Book chapter	6	1.4
Meeting abstract	4	1
Short survey	1	0.2
Erratum	1	0.2
Correction	1	0.2
Total	444	100

**Table 2:** The first 10 authors were ranked by number of papers.

Rank	Author	Country	Number of Papers	Citations	Total Link Strength
1	Ordonez F.J.	Spain	20	177	62
2	Rosety R.M.	Spain	20	177	62
3	Fernhall B.	USA	18	578	38
4	Ptomey L.T.	USA	11	28	49
5	Shields N.	Australia	10	606	9
6	Izquierdo G.R.	Spain	9	180	31
7	Veiga O.L.	Spain	9	180	30
8	Rosety I.	Spain	9	69	48
9	Fornieles G.	Spain	9	62	49
10	Donnelly J.E.	USA	9	27	44

**Table 3:** Top 20 keywords ranked by co-occurrences with the two keywords “Down syndrome” and “physical activity”.

Rank	Keyword	Occurrences
1	Adolescent	45
2	Obesity	39
3	Intellectual disability	29
4	Body composition	28
5	Activities of daily living	27
6	Developmental disability	22
7	Cognitive function	20
8	Cardiovascular system	19
9	Balance	17
10	Gait analysis	16
11	Dietary habit	15
12	Muscle mass	14
13	Accelerometry	13
14	Metabolism	12
15	Neurodegenerative disease	12
16	Rehabilitation	12
17	Posture	12
18	Oxidative stress	12
19	Disabled	10
20	Fat mass	10

**Table 4:** Top 20 co-occurring keywords with the keywords “Down syndrome” and “physical activity” in the literature, published by decade (2001 to 2009, 2010 to 2019, 2020 to 2024).

	2001 to 2009		2010 to 2019		2020 to 2024	
Rank	Keyword	Occurrences	Keyword	Occurrences	Keyword	Occurrences
1	Oxidative stress	8	Adolescent	22	Adolescent	22
2	Antioxidant	6	Obesity	15	Obesity	21
3	Rehabilitation	5	Body composition	13	Dietary habit	14
4	Intellectual disability	5	Cardiovascular system	12	Developmental disability	14
5	Metabolism	4	Intellectual disability	11	Intellectual disability	13
6	Body composition	4	Activity of daily living	11	Activity of daily living	12
7	Activity of daily living	4	Cognitive function	9	Body composition	11
8	Hormone	4	Balance	8	Posture	11
9	Cardiovascular system	3	Developmental disability	7	Cognitive function	11
10	Obesity	3	Bone mineral density	7	Gait analysis	10
11	Gait analysis	3	Accelerometry	7	Neurodegenerative disease	9
12	Cardiovascular disease	2	Barrier	6	Muscle mass	8
13	Disabled	2	Blood pressure	6	Balance	8
14	VO2max	2	Cardiovascular disease	6	Sedentary behavior	7
15	Fat mass	2	Facilitator	5	Accelerometry	6
16	Infant	2	Muscle mass	5	Disabled	6
17	Motor coordination	2	Respiration	5	Parent	6
18	-	-	Female	4	Fat mass	5
19	-	-	Early intervention	4	Cardiovascular system	5
20	-	-	Metabolism	4	Metabolism	4

**Table 5:** Physiotherapy Evidence Database (PEDro) scale of the studies included in the meta-analysis.

Study	1	2	3	4	5	6	7	8	9	10	11	PEDro Score (0–10)
Varela et al. (2001) [34]	Y	Y	N	Y	N	N	N	Y	N	Y	Y	5
Casey & Emes (2011) [35]	Y	N	N	Y	N	N	N	Y	N	Y	Y	4
Mendonca et al. (2011) [36]	Y	Y	N	Y	N	N	N	Y	N	Y	Y	5
Ordonez et al. (2012) [37]	Y	Y	N	Y	N	N	N	Y	N	Y	Y	5
Rosety-Rodriguez et al. (2014) [38]	Y	Y	N	Y	N	N	N	Y	N	Y	Y	5
Seron et al. (2014) [39]	Y	N	N	Y	N	N	N	Y	N	Y	Y	4
Ordonez et al. (2014) [40]	Y	Y	Y	Y	N	N	Y	Y	N	Y	Y	7
Shields & Taylor (2015) [41]	Y	Y	Y	Y	N	N	Y	Y	Y	Y	Y	8
Silva et al. (2017) [42]	Y	Y	N	Y	N	N	Y	Y	N	Y	Y	6
Ptomey et al. (2018) [43]	Y	Y	Y	Y	N	N	Y	Y	Y	Y	Y	8
Boer & de Beer (2019) [44]	Y	N	N	Y	N	N	N	Y	N	Y	Y	4
Suarez-Villadat et al. (2020) [45]	Y	Y	N	Y	N	N	Y	Y	N	Y	Y	6
Boer (2020) [46]	Y	Y	N	Y	N	N	Y	Y	Y	Y	Y	7
Naczk et al. (2021) [47]	Y	Y	N	Y	N	N	Y	Y	N	Y	Y	6
Suarez-Villadat et al. (2023) [48]	Y	Y	Y	Y	N	N	Y	Y	Y	Y	Y	8

N: no, does not meet the criteria; Y: yes, meets the criteria. The PEDro scale criteria are (1) eligibility criteria; (2) random allocation; (3) concealed allocation; (4) groups similar at baseline; (5) blinding of participants; (6) blinding of therapists; (7) blinding of assessors; (8) adequate follow-up (<15% dropouts); (9) intention-to-treat analysis; (10) between-group comparisons; and (11) point estimates and variability. (1) eligibility criteria is not included in total score ranging 0 to 10.

**Table 6:** Characteristics of the included studies for the meta-analysis.

Study (Year, Country)	N (IG/CG)	Age (Years)	Gender (M/F)	Duration (Week) (Frequency)	Intervention	CG
Varela et al. (2001, Portugal) [34]	16 (8/8)	IG 22.0 ± 3.8 CG 20.8 ± 2.3	16/0	16 (3×/week)	Rowing ergometer training - Weeks 1–4: 55–60% VO_2_peak - Weeks 5–16: 70% VO_2_peak	-
Casey et al. (2011, Canada) [35]	20 (10/10)	IG: 23.1 ± 4.2 CG: 22.8 ± 3.9	11/9	10 (3×/week)	Progressive RT + AT - RT: Leg Press, Leg Curl, Chest Press, Shoulder Press, Lat Pulldown (8~15 reps × 3 sets) - AT: Treadmill/Ergometer (50~70% HRmax)	-
Mendonca et al. (2011, Portugal & USA) [36]	25 (13/12)	IG: 36.5 ± 5.5 CG: 38.7 ± 8.3	19/6	12 (3×/week)	Progressive RT + AT - RT: Leg Press, Chest Press, Lat Pulldown, Shoulder Press, Lower Back Extension, Leg Extension, Biceps Curl, Triceps Pushdown, Abdominal Curls (14 reps × 2 sets) - AT: Treadmill (65~85% VO_2_peak)	-
Ordonez et al. (2012, Spain) [37]	20 (11/9)	IG: 24.7 ± 3.6 CG: 25.1 ± 3.9	0/20	10 (3×/week)	AT - Treadmill (55~65% HRmax)	-
Rosety-Rodriguez et al. (2014, Spain) [38]	20 (11/9)	IG: 24.7 ± 3.6 CG: 25.1 ± 3.9	0/20	10 (3×/week)	AT - Treadmill (55~65% HRpeak)	-
Seron et al. (2014, Brazil) [39]	26 (16/10)	IG: 15.7 ± 2.7 CG: 14.4 ± 2.5	10/16	12 (3×/week)	AT - Treadmill/Ergometer (55~65% HRR)	-
Ordonez et al. (2014, Spain) [40]	20 (11/9)	IG: 24.7 ± 3.6 CG: 25.1 ± 3.9	0/20	10 (3×/week)	AT - Treadmill (55~65% HRpeak)	-
Shields & Taylor, (2015, Australia) [41]	16 (8/8)	IG: 21.6 ± 3.4 CG: 21.2 ± 3.2	8/8	8 (3×/week)	AT - Walking (45–60 min)	-
Silva et al. (2017, Brazil) [42]	25 (12/13)	18–60	N/A	8 (3×/week)	Multi-component exercise program - Balance: Wii Fit Balance Board - AT: Wii Sports, Wii Sports Resort, Wii Fit (swordplay, boxing, cycling, table tennis)	-
Ptomey et al. (2018, USA) [43]	124 (21/103)	IG: 36.5 ± 9.6 CG: 36.5 ± 12.5	77/54	24 (5×/week)	Walking program - 30 min/day at moderate intensity	-
Boer & de Beer, (2019, South Africa) [44]	23 (13/10)	31.4 ± 7.4	13/10	8 (3×/week)	Aquatic exercise - 35–45 min per session	-
Suarez-Villadat et al. (2020, Spain) [45]	45 (15/30)	EG: 13.93 ± 1.23 CG: 13.71 ± 1.24	25/20	36 (3×/week)	Swimming program - Front Crawl: 160–180 bpm - Breaststroke: 110–130 bpm	Recreational swimming of 300–400 m per session (2×/week)
Boer (2020, South Africa) [46]	26 (13/13)	IG: 33 ± 6 CG: 33 ± 6	13/13	8 (3×/week)	Freestyle Swim Training - Water walking, arm swings, stretching, high intensity running on the spot, lunge jumps, squat jumps, flutter kicks, repetitive freestyle swim training, swimming with a kickboard (30–40 min)	-
Naczk et al. (2021, Poland) [47]	22 (11/11)	IG: 14.9 ± 2.35 CG: 14.4 ± 1.97	14/8	33 (3×/week)	Swimming and Aquatic exercise - Weeks 1–4: Adaptation Phase - Weeks 5–8: Basic Skills Phase - Weeks 9–23: Skill Acquisition Phase - Weeks 24–33: Proficiency Phase	-
Suarez-Villadat et al. (2023, Spain) [48]	49 (24/25)	14.19 ± 2.06	30/19	20 (3×/week)	Multi-component exercise program - Wii Sports, Wii Sports Resort (i.e., tennis, baseball, bowling, golf, canoe, cycling, boxing)	A physical activity program based on developing motor behavior (3×/week) was performed

N, the number of subjects; IG, intervention group; CG, control group; M, male; F, female; RT, resistance training; AT, aerobic training; HRR, heart rate reserve; Data for age are mean ± standard deviation.

## Data Availability

The review protocol for meta-analysis was registered at PROSPERO: “https://www.crd.york.ac.uk/PROSPERO/view/CRD420261304004 (accessed on 8 February 2026)”.

## Supplementary Materials

The following supporting information can be downloaded at: https://www.mdpi.com/article/10.3390/healthcare14081052/s1, Figure S1: Assessment of publication bias using funnel plot symmetry and Egger’s regression test. The funnel plot displays the relationship between study-specific effect sizes and their standard errors for all included studies. In the absence of publication bias, the distribution of studies is expected to be symmetrical around the pooled effect estimate, whereas asymmetry may indicate potential small-study effects. Visual inspection of the funnel plot suggested [symmetry/asymmetry], indicating [low/potential] risk of publication bias. This was formally evaluated using Egger’s regression test, which assesses the association between effect sizes and their precision. Our test results suggest that body weight, body mass index (BMI), fat mass, waist circumference (WC) are robust, while waist-to-hip ratio (WHR) is potentially influenced by publication bias.

## Abbreviations

The following abbreviations are used in this manuscript:

DS	Down syndrome
PA	Physical activity
AT	Aerobic training
BW	Body weight
BMI	Body mass index
FM	Fat mass
WC	Waist circumference
WHR	Waist-to-hip ratio
SMD	Standardized mean difference
RCT	Randomized controlled trial
PEDro	Physiotherapy evidence database
