# Supplementary material for: Aerobic Training for Obesity Management in Individuals with Down Syndrome: A Bibliometric and Meta-Analyses
Source: Healthcare (Basel). 2026 Apr 15;14(8):1052. doi: 10.3390/healthcare14081052 (PMC13116952; doi:10.3390/healthcare14081052)
Supplement: Supplementary file 1 [file healthcare-14-01052-s001.zip › healthcare-4198882-supplementary.pdf]

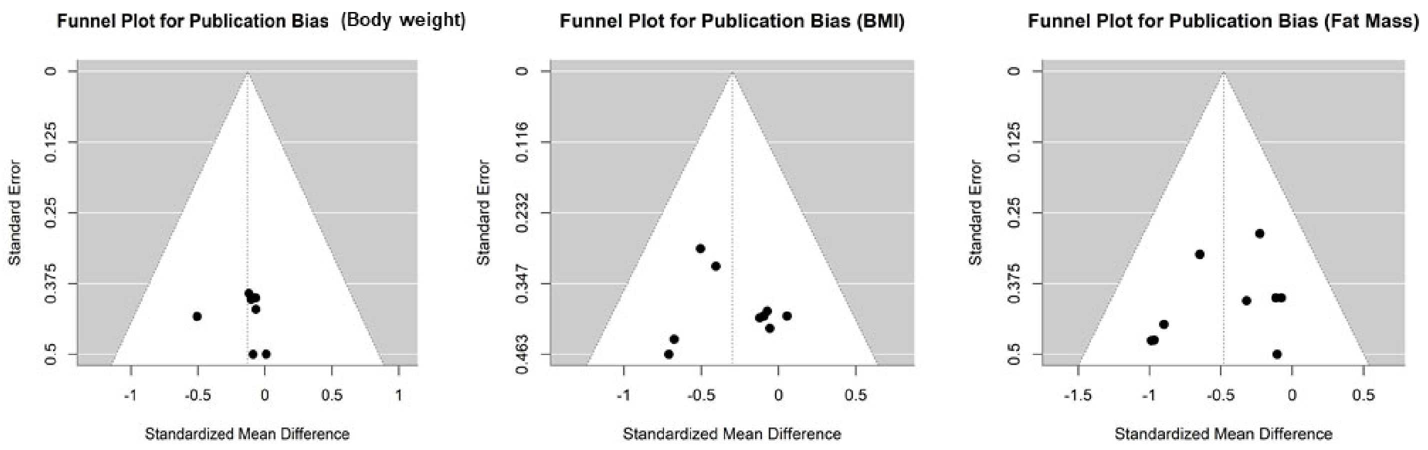

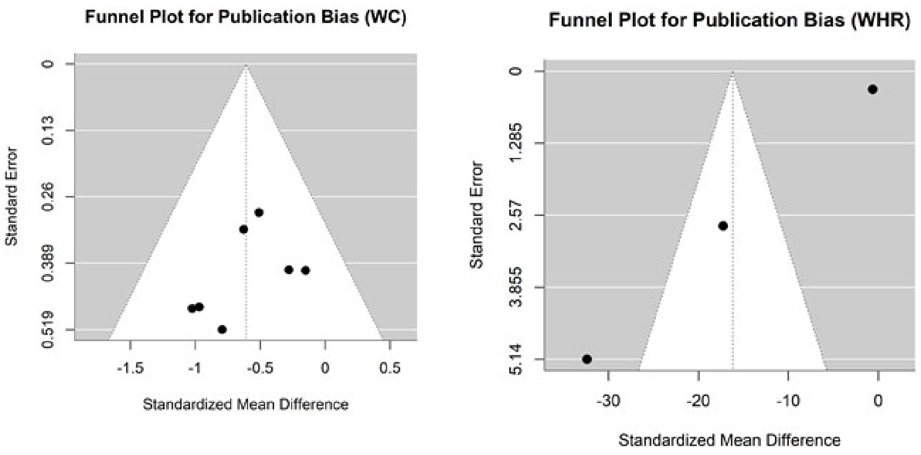

Egger's test

Variables	β	P
Body weight	-0.64	0.60
BMI	-0.22	0.89
Fat mass	0.3	0.24
WC	0.09	0.21
WHR	1.54	0.01

Supplementary Figure S1. Assessment of publication bias using funnel plot symmetry and Egger's regression test. The funnel plot displays the relationship between study-specific effect sizes and their standard errors for all included studies. In the absence of publication bias, the distribution of studies is expected to be symmetrical around the pooled effect estimate, whereas asymmetry may indicate potential small-study effects. Visual inspection of the funnel plot suggested [symmetry/asymmetry], indicating [low/potential] risk of publication bias. This was formally evaluated using Egger's regression test, which assesses the association between effect sizes and their precision. Our test results suggest that body weight, body mass index (BMI), fat mass, waist circumference (WC) are robust, while waist-to-hip ratio (WHR) is potentially influenced by publication bias.
